# Prevalence of central sleep apnea among veterans and response rate to continuous positive airway pressure therapy

**DOI:** 10.1093/sleepadvances/zpae011

**Published:** 2024-02-05

**Authors:** Nesrine Adly Ibrahim, Abdulghani Sankari, Ahmad Aldwaikat, Nishtha Pandya, Susmita Chowdhuri, Anan Salloum, Jennifer L Martin, Salam Zeineddine, M Safwan Badr

**Affiliations:** Department of Medicine, John D. Dingell VA Medical Center, Detroit, MI, USA; Department of Internal Medicine, Wayne State University, Detroit, MI, USA; Department of Medicine, John D. Dingell VA Medical Center, Detroit, MI, USA; Department of Internal Medicine, Wayne State University, Detroit, MI, USA; Department of Medical Education, Ascension Providence Hospital, Southfield, MI, USA¸; Department of Medicine, John D. Dingell VA Medical Center, Detroit, MI, USA; Department of Internal Medicine, Wayne State University, Detroit, MI, USA; Department of Medicine, John D. Dingell VA Medical Center, Detroit, MI, USA; Department of Internal Medicine, Wayne State University, Detroit, MI, USA; Department of Medicine, John D. Dingell VA Medical Center, Detroit, MI, USA; Department of Internal Medicine, Wayne State University, Detroit, MI, USA; Department of Medicine, John D. Dingell VA Medical Center, Detroit, MI, USA; Department of Internal Medicine, Wayne State University, Detroit, MI, USA; Department of Medicine, David Geffen School of Medicine at the University of California, Los Angeles, CA, USA; Geriatric Research, Education and Clinical Center, VA Greater Los Angeles Healthcare System, Los Angeles, CA, USA; Department of Medicine, John D. Dingell VA Medical Center, Detroit, MI, USA; Department of Internal Medicine, Wayne State University, Detroit, MI, USA; Department of Medicine, John D. Dingell VA Medical Center, Detroit, MI, USA; Department of Internal Medicine, Wayne State University, Detroit, MI, USA

**Keywords:** central sleep apnea, sleep-disordered breathing, PAP therapy

## Abstract

**Study Objectives:**

Sleep-disordered breathing (SDB) is common in the Veteran population. In this retrospective study, we investigated the prevalence of comorbid central and obstructive SDB and the response rate to PAP among Veterans.

**Methods:**

Veterans were screened from a single VA medical center who had polysomnography (PSG) study from 2017 to 2021 to ascertain the presence, severity, and type of SDB by measuring the apnea–hypopnea index (AHI) and central apnea index (CAI). Patients were excluded if they did not have complete studies (diagnostic and PAP titration studies). The inclusion criteria for these analyses were central sleep apnea (CSA) defined as AHI ≥ 10 events/hour and CAI ≥ 5 events/hour. Diagnostic “CSA only” was defined as AHI ≥ 10 events/hour and CAI ≥ 50% of AHI. “OSA only” was defined if AHI ≥ 10 events/hour and CAI < 5 events/hour. Comorbid central and obstructive sleep apnea (COSA) was defined if AHI ≥ 10 events/hour and CAI > 5 events/hour but < 50% of AHI. The responsiveness to PAP therapy was determined based on the CAI < 5 events/hour on the titration study.

**Results:**

A total of 90 patients met the inclusion criteria and from those 64 Veterans were found to have COSA (71%), 18 (20%) were CSA only, and 8 (9%) were OSA only. A total of 22 (24.4%) Veterans diagnosed with CSA or COSA were responsive to PAP therapy. Sixty days after treatment initiation, both responsive and nonresponsive groups had significant decreases in AHI and CAI (*p* < 0.05).

**Conclusions:**

Comorbid central and obstructive SDB is common among Veterans. The response to PAP therapy is suboptimal but improves over time.

**Statement of Significance**

Sleep-disordered breathing (SDB) is highly prevalent among Veterans. Patients with central sleep apnea (CSA) frequently commonly experience fatigue, morning headaches, and excessive sleepiness. CSA is often difficult to manage, which can lead to serious long-term health issues such as hypertension, arrhythmia, diabetes mellitus, hypercholesterolemia, and cognitive impairment. The objective of this study is to provide preliminary evidence on the impact of central or comorbid central and obstructive SDB among Veterans, and the effectiveness of the use of PAP therapy. Our findings confirm that most Veterans have comorbid central and obstructive sleep apnea (COSA) and more than half of them are not responsive to PAP initially, but they respond with long-term use.

## Introduction

Sleep-disordered breathing (SDB) encompasses multiple forms of unstable breathing during sleep, including obstructive sleep apnea (OSA), central sleep apnea (CSA), and mixed sleep apnea. SDB is common among Veterans, estimated at up to 22% [1], in comparison to the general population which accounts for 17.4%–3.9% in women and men, respectively [2]. OSA is the most common form in the general population [3, 4]. However, CSA may coexist with OSA and complicate disease management. A minority of Veterans who have SDB have isolated CSA (2%) although this rate may be increasing over time [5–7]. The prevalence of comorbid central and obstructive sleep apnea (COSA) among Veterans remains unknown.

Currently, positive pressure therapy (PAP) is the treatment of choice for SDB-central and obstructive types; however, PAP therapy may be challenging in Veterans, given the multitude of comorbid conditions that increase susceptibility to CSA, including opioid use, heart failure [8], in addition to comorbid insomnia and post-traumatic stress disorder [9]. In addition, previous studies demonstrated that CSA presence among Veterans lowered response to PAP therapy to less than 50% [10]. Therefore, we sought to determine the response to PAP therapy in Veterans diagnosed with CSA and comorbid COSA. We hypothesized that PAP therapy would be efficacious in Veterans who have CSA and among those with COSA.

## Materials and Methods

We retrospectively reviewed the Veterans Health Administration (VA) computerized patient record system for consecutive Veterans who were referred to the John D. Dingell VA Sleep Disorders Center and carry the diagnosis of CSA (using the following ICD-10 codes: G47.30, G47.31, G47.37, and G47.39) (Supplementary Appendix 1) between January 2017 and December 2021. All participants completed polysomnography (PSG) to measure the apnea–hypopnea index (AHI) and central apnea index (CAI), which was used to assess the presence and severity of sleep apnea. The Institutional Review Boards of Wayne State University of Medicine and the Detroit Veterans Affairs Medical Center approved the study protocol.

The inclusion criterion was patients with SDB (AHI ≥ 10 events/hour). Participants were excluded if they did not have complete studies (both diagnostic and titration studies) and if the was an AHI of less than 10 events/hour. CSA was defined as AHI ≥ 10 events/hour and CAI ≥ 50% of AHI. OSA was defined as AHI ≥ 10 events/hour and CAI < 5 events/hour. COSA was defined if AHI ≥ 10 events/hour and CAI > 5 events/hour but < 50% of AHI. We defined “responsive to PAP” if AHI was < 5/hour on the PAP.

### Data analysis

The data elements used in these analyses were extracted from the electronic health record. For all included Veterans we recorded the demographic data, clinical diagnoses, sleep parameters during the diagnostic study and initial PAP titration (PAP0), and adherence to PAP therapy at day 60 days after PAP initiation (PAP60).

#### Demographics and clinical diagnoses.

All demographic and diagnostic data were extracted manually through the VA electronic health record computerized patient record system and data were de-identified.

### Sleep measures

The diagnostic study consisted of overnight polysomnography (PSG) (SomnoStar 10.2) to assess the presence and severity of SDB based on the AHI. Data from the electrooculogram, electroencephalogram, electrocardiogram, electromyogram, airflow measurement, and pulse oximeter were recorded. Respiratory events were scored and reviewed by a board-certified sleep physician using the American Academy of Sleep Medicine (AASM) scoring manual [11]. Scoring of the respiratory events was based on the following criteria: apneas were defined as the cessation of airflow for 10 seconds or longer with the presence of respiratory efforts (obstructive apneas) or absence of respiratory efforts (central apneas). Hypopneas were defined according to AASM recommended criteria as a reduction in airflow (≥30%) for 10 seconds or longer, associated with either a ≥ 3% oxygen desaturation or an arousal. AHI was the number of events (apneas and hypopneas) per 1 hour of sleep. CAI was of the number of central respiratory events per hour of sleep. PAP was titrated per AASM guidelines to achieve optimal pressure that eliminated respiratory events using two types of PAP devices (CPAP and BPAP) [12]. Oxygen was added according to a previously published CSA treatment protocol, where supplemental O2 was added at 2 L/min and increased by 1 L/min to maintain oxygen saturation ≥ 93%, keeping PAP at the same level [13]. Residual AHI and CAI were collected during the initial PAP titration night (PAP0) using the number of apnea and hypopnea per hour of sleep on therapeutic PAP level and after 60 days of PAP use (PAP60) using average AHI and CAI from days PAP used over 60 days.

### PAP adherence

Adherence to PAP therapy was measured by Respironics online monitoring program (EncoreAnywhere™) or ResMed online mentoring program (myAir™) depending upon the PAP device dispensed to the patients. Adequate adherence was defined as PAP usage > 4 h/night for 70% of the days [14].

### Statistical analysis

Descriptive statistics were computed for all study variables (mean/standard deviation). A Student *t*-test was used to compare sleep variables between the PAP-responsive versus PAP-nonresponsive groups. In our analysis, all the variables in the dataset failed the Shapiro–Wilk test of normality, indicating a departure from normal distribution. To investigate the significance of differences in the median across various groups’ measurements, we performed a Kruskal–Wallis One-Way Analysis of Variance on the Ranks test. Subsequently, post hoc analyses using Dunn’s Method were conducted to identify specific group differences.

Furthermore, we conducted a Spearman correlation analysis to examine the relationships between various variables. Then a multilinear regression model was used on variables that had *p*-values < 0.05. For each test, *p* < 0.05 was considered statistically significant. Analyses were conducted using SPSS and SigmPlot 11.0.

## Results

**Figure 1.**
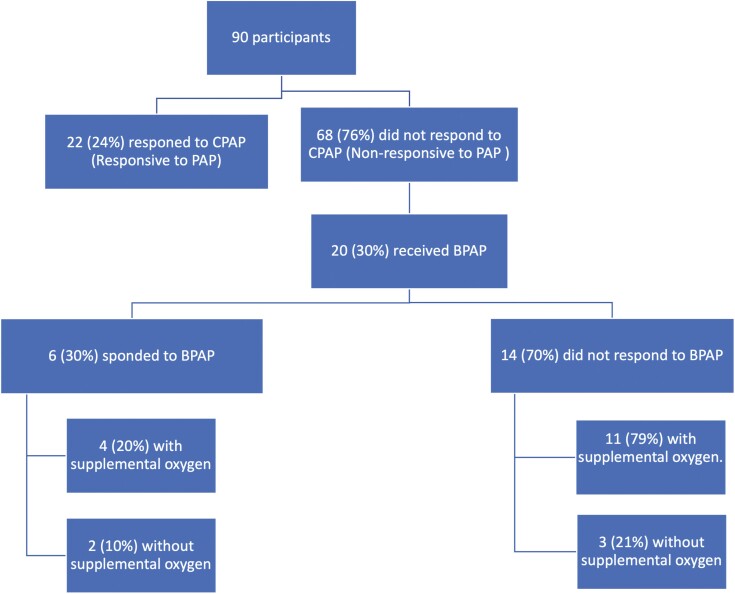
Flow chart illustrating the number of responsive and nonresponsive to initial PAP titration and progress of therapy. BPAP, bilevel positive pressure therapy; CPAP, continuous positive airway pressure; PAP, positive pressure therapy.

**Figure 2.**
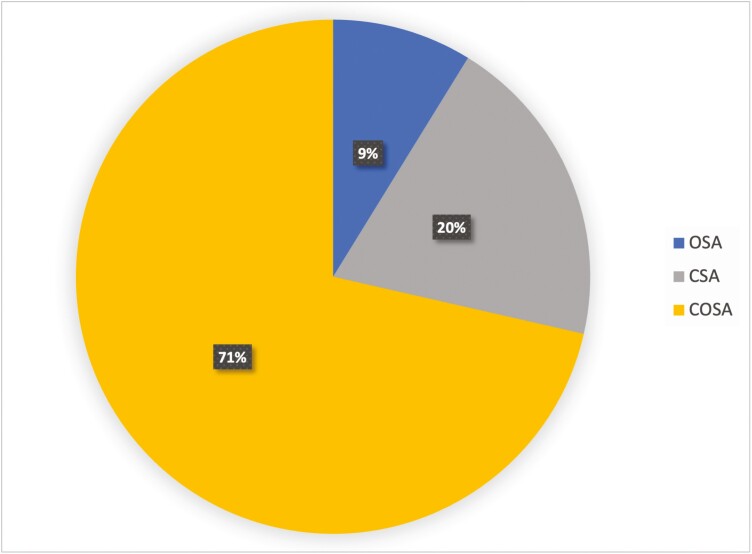
Pie chart illustrating the percentage of occurrence of isolated CSA, isolated OSA, and COSA. CSA, central sleep apnea; OSA, obstructive sleep apnea; COSA, comorbid central and obstructive sleep apnea.

Characteristics of the study sample are shown in Table 1. There was no significant difference in demographic or polysomnographic data between the PAP responsive and nonresponsive groups, other than the significant decrease in AHI and CAI in the PAP titration study compared to the AHI and CAI in the diagnostic study (*p* < 0.05). However, residual AHI and CAI were not significantly different between the two groups at day 60 of PAP use. Figure 1 illustrates the number of patients who responded or did not respond to PAP and their progression in treatment including those who needed BPAP and/or oxygen supplementation. None of the patients had adaptive servo-ventilation. Figure 2 illustrates the proportions of each type of SDB with the most to least common being comorbid COSA, CSA, and OSA, respectively. Summary data specific for each SDB type are outlined in Supplementary Appendix 2.

**Table 1.** Characteristics of Study Sample

Characteristics	Total	Nonresponsive to PAP	Responsive to PAP	Responsive versus nonresponsive (*P*-value)
*N*	90	68	22	
Age (y)	66.0 ± 12.7	66.4 ± 13.1	64.5 ± 11.9	0.557
Gender (M/F)	88/3	67/1	20/2	0.793
BMI (Kg/m^2^)	30.7 ± 5.5	30.8 ± 5.2	30.8 ± 6.0	0.967
OSA only (*N*, %)	9 (9)	4 (6)	4 (18)	0.079
CSA only (*N*, %)	18 (20)	15 (22)	3 (14)	0.396
COSA (*N*, %)	64 (71)	49 (72)	15 (68)	0.730
Diagnostic AHI (event/hour)	75.4 ± 24.7	74.9 ± 22.2	73.9 ± 29.0	0.858
Diagnostic CAI (event/hour)	24.0 ± 23.1	25.8 ± 24.5	18.5 ± 18.1	0.206
Diagnostic HI (event/hour)	34.3 ± 22.0	32.3 ± 20.7	38.0 ± 22.7	0.283
Diagnostic OAI (event/hour)	11.4 ± 15.8	11.0 ± 16.7	11.7 ± 12.5	0.860
Diagnostic nadir O_2_ (%)	82.7 ± 6.8	84.3 ± 5.8	82.1 ± 7.4	0.217
Diagnostic %O_2_ saturation <90 (%)	82.7 ± 6.8	29.0 ± 28.6	36.6 ± 32.3	0.295
AHI on final PAP level during titration study (PAP0) (event/h)	30.1 ± 29.7	39.0 ± 29.1	2.7 ± 1.5	<0.001
CAI on final PAP level during titration study (PAP0) (event/h)	14.9 ± 22.7	19.4 ± 24.4	0.7 ± 1.2	0.001
Received O_2_ treatment with PAP (*N*, %)	22 (24)	22 (32%)	0 (0)	0.001

AHI, apnea–hypopnea index; BMI, body mass index; CAI, central apnea index; COSA, comorbid obstructive and central sleep apnea; CSA, central sleep apnea; HI, hypopnea index; OAI, obstructive apnea index; PAP, positive airway pressure; PAP0, PAP titration study; OSA, obstructive sleep apnea.

**Figure 3.**
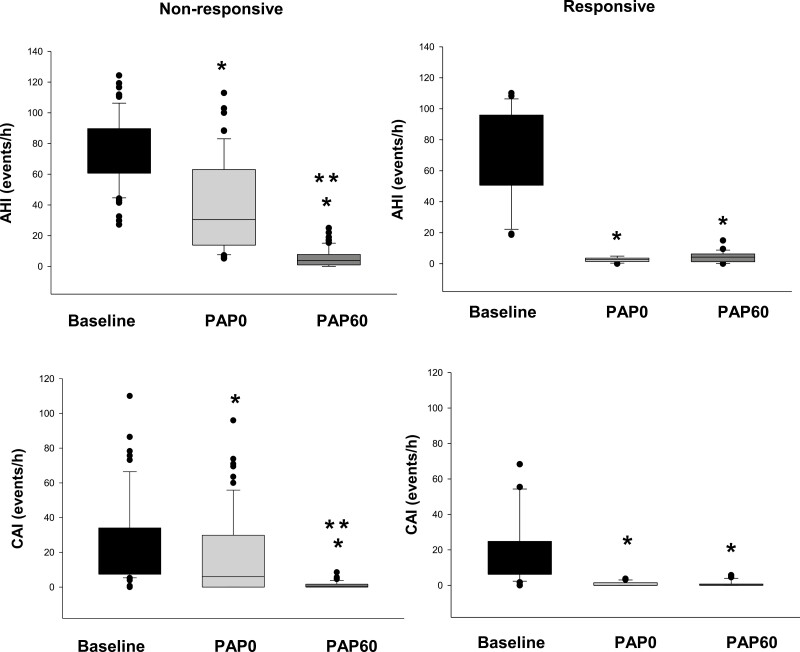
Boxplots illustrating the comparison between AHI and CAI in diagnostic, titration (PAP 0), and 60 days after PAP use (PAP 60). CAI, central apnea index, AHI, apnea–hypopnea index; PAP, positive pressure therapy. * Indicates *p* < 0.05 versus Diagnostic using repeated measure ANOVA on rank and Dunn’s test for pairwise comparisons. ** Indicates *p* < 0.05 versus PAP titration (PAP 0) using repeated measure ANOVA on rank and Dunn’s test for pairwise comparisons.

In Figure 3, we focused on nonresponsive participants and compared the diagnostic, titration, and PAP for 60-day levels of AHI and CAI measurements. Interestingly, we found no significant difference in median titration and median PAP for 60 days of both AHI and CAI levels in the responsive group. However, a significant difference was observed in the median diagnostic and median PAP for 60 days for both AHI and CAI levels in the nonresponsive group. However, despite the significant decrease in AHI and CAI in both the responsive and nonresponsive groups between initial PAP and day 60, 32 of the 57 (56%) of the nonresponsive group had residual AHI < 5 events/hour at day 60 and 9 of the 21 (43%) of the responsive group had AHI < 5 events/hour as shown in Table 2 (*p* = 0.30). Notably, both responsive and nonresponsive groups had modest and similar levels of adherence to PAP both by days used and traditional threshold of at least 4 hours use or more for more than 70% of the nights (49.3 and 34.3 %, respectively, *p* = 0.118). As outlined in Table 3, no significant difference was found between those who had optimal adherence to PAP and those who did not have optimal adherence. Likewise, no significant difference was found between those who had adherence data available and those who did not have data on download except for AHI and CAI at baseline as shown in Supplementary Appendix 3.

**Table 2.** Response to PAP Therapy and Adherence at Day 60

Characteristics	Total (*n* = 78)	Nonresponsive to PAP (*n* = 57)	Responsive to PAP (*n* = 21)	Responsive versus nonresponsive (*P*-value)
Residual AHI on PAP at day 60 (event/hour)	6.1 ± 5.4	6.7 ± 5.9	4.6 ± 3.5	0.125
Residual CAI on PAP at day 60 (event/hour)	1.3 ± 1.8	1.5 ± 1.8	0.8 ± 1.5	0.166
Total days used (%) at day 60	58.1 ± 38.2	52.2 ± 37.3	63.7 ± 37.6	0.237
PAP use >4 hours/day (%) at day0	44.7 ± 37.6	34.3 ± 34.3	49.3 ± 38.1	0.118

AHI, apnea–hypopnea index; CAI, central apnea index; PAP, positive airway pressure.

**Table 3.** Characteristics of the Study Sample Based on Adherence to PAP

Characteristics	Adherent	Non-adherent
*N*	30	48
Age (y) Gender (M/F)	68.3 ± 9.9 30/0	65.7 ± 12.5 45/3
BMI (Kg/m^2^)	32.3 ± 4.2	30.3 ± 5.7
Diagnostic AHI (event/hour)	80.0 ± 18.3	72.4 ± 26.9
Diagnostic C)	20.6 ± 18.6	24.1 ± 24.1
Diagnostic HI (event/hour)	35.0 ± 20.7	33.4 ± 20.8
Diagnostic OAI (event/hour)	17.8 ± 16.8	9.0 ± 15.1^*^
AHI on final PAP level during titration study (PAP0) (event/hour)	27.1 ± 24.8	24.2 ± 28.0
CAI on final PAP level during titration study (PAP0) (event/hour)	11.7 ± 19.4	11.0 ± 18.0

AHI, apnea–hypopnea index; BMI, body mass index; CAI, central apnea index; HI, hypopnea index; OAI, obstructive apnea index; PAP, positive airway pressure; PAP0, PAP titration study.

^*^Indicate *p* < 0.05 versus adherent non-paired Student *t*-test.

Lastly, we investigated the correlation between the initial response to PAP (AHI on PAP titration night) and variables of interest such as diagnostic AHI, diagnostic CAI, age, and BMI. Specifically, we observed a significant correlation between AHI on PAP for 60 days and diagnostic CAI, diagnostic AHI and diagnostic CAI, diagnostic AHI, and PAP Pressure, as well as between BMI and Age. We found that there is a significant positive correlation between AHI response on PAP0 and diagnostic CAI (coefficient was 0.28 *p* < 0.01). Using Multiple Linear Regression, the initial response to PAP was predicted from diagnostic CAI (*R* = 0.34 and *p* < 0.01). On day 60, however, no correlation was found between residual AHI-day 60 and any of the following variables: diagnostic AHI, diagnostic CAI, age, and BMI (*p* = NS).

## Discussion

Our study demonstrates the following novel findings: (1) Isolated central apnea is uncommon among Veterans. Comorbid central and obstructive apnea (COSA) is common among Veterans diagnosed with CSA., (2) Adherence to PAP therapy in this population is below 50%., (3) Initial response rate to PAP therapy is low (~25%) despite supplemental Oxygen (O_2_) therapy in a third of the sample., (4) Continued use of PAP therapy for 2 months was associated with significant improvement in response to PAP; however, a significant number of patients (~40%) continued to experience high residual COSA (AHI≥ 5 events/hour). Accordingly, the efficacy of PAP in reducing AHI is partial.

CSA is uncommon in the general population as the diagnostic criteria require that only patients with at least 50% central events can be diagnosed with CSA [6]. Distinguishing central from obstructive events is rooted in morphology, not pathophysiology [15–18]. CSA and OSA are intertwined physiologically, as evidenced by upper airway narrowing or occlusion during central apneas [19] and hypopneas [15], upper airway obstruction at the nadir of periodic breathing, and a reversible increase in propensity to CSA in patients with OSA [20]. Our findings support the coexistence of both types of events in our population.

The poor adherence to PAP therapy, while disappointing, is not unexpected and is in line with adherence to therapy in chronic health conditions such as pharmacologic treatment of hypertension [21]. It appears that challenges with the use of PAP therapy apply to patients with CSA and COSA similar to the challenges experienced by patients with isolated OSA [22]. Although it is unclear which group benefits long-term from treatment, especially mild disease.

The poor initial response to PAP in most patients with CSA is not surprising as several previous reports indicated similar findings [10, 23, 24]. Interestingly, the poor response also occurred in the COSA group. This initial poor response to PAP was predicted by diagnostic CAI indicating the importance of underlying CSA in the response rate in COSA; further, this may suggest that CSA could be a marker of poor response to PAP therapy. In patients with SDB. Interestingly, we noted a significant decrease in AHI compared to the PAP titration night after 60 days of PAP therapy. Decreased CSA severity with PAP therapy in our study is analogous to the resolution of treatment-emergent CSA (TECSA) [25], improvement in CSA propensity in patients with OSA [20], and decreased hypoxic ventilatory response following PAP therapy [20, 26]. Nevertheless, four out of ten patients continue to have AHI above 5 events/hour at 60 days of therapy, suggesting that the underlying mechanism may not be fully reversible.

### Clinical implications

This study provides several clinical implications. First, COSA is a unique type of SDB and is not a new clinical phenomenon but is under-recognized due to limitations of the current definition of CSA. The use of restrictive criteria for the diagnosis of CSA may impede access to specific CSA treatments and may also affect the feasibility and generalizability of clinical trials investigating CSA. Furthermore, restrictive CSA diagnostic criteria may amplify disparity in the diagnosis and management of CSA in women given the paucity of CSA in premenopausal women [27], and in individuals with an unfavorable upper airway due to obesity or increased intravascular volume in HF [28]. Inclusion of all patients with CSA—including those with comorbid OSA—will enhance the feasibility, generalizability, and ecological validity of research addressing CSA treatment. Clinicians should be aware of this condition given that the majority do not respond initially to treatment.

Second, the significant decrease in residual AHI after 60 days of treatment is important therapeutically. The magnitude of response to PAP therapy can be predicted from the severity of CSA during the diagnostic PSG study. Third, despite a significant decrease in the severity of residual AHI after 60 days of PAP therapy more than a third of patients continue to have elevated residual AHI (>5 events/hour). However, the clinical implication of this residual disease is unknown.

### Methodological considerations

Several limitations to this study should be considered. First, the sample size is relatively small and from one single VA medical Center, and very few women are included in the sample. Likewise, race and ethnic data were not reliably available in the medical record and hence were not included in this study. Therefore, larger studies are needed to generalize these interesting findings. Second, the sleep studies were scored according to the AASM manual; however, subclassifications of hypopnea to obstructive or central were not performed as this was part of the clinical record of patients scored previously by board-certified sleep physicians. Therefore, the reported AHI may underestimate the severity of central SDB if there are a significant number of central hypopneas. Third, the follow-up of the study was for 60 days which was thought to be a reasonable period for initial assessment of adherence immediately after the initiation of treatment, therefore it is unknown if the additional clinical response would occur beyond the 60 days. Future studies could extend the monitoring to 6–12 months to assess the clinical response to PAP therapy and residual AHI. Fourth, details on comorbidities such as cardiac function to compare the groups were not available at the time of the study. Fifth, the threshold for defining SDB and choosing a threshold of AHI above 10 events/hour was to allow the ability to capture mild CSA as we wanted to have CAI with at least 5 events/hour. This could miss those with milder disease (i.e. AHI 5–10 events/hour) who may also have CSA. Finally, the follow-up assessment at day 60 (PAP60) is based on PAP download data, which is different from the sleep study and initial PAP titration (PAP0), hence there is a potential limitation in comparing AHI between these settings that should be considered.

## Conclusion

In conclusion, COSA is a common condition among US veterans. Many patients do not respond initially to PAP therapy but improve after 60 days of treatment. Therefore, alternative therapies or combined therapeutic options are warranted.

## Supplementary Material

zpae011_suppl_Supplementary_Appendix
