# Supplementary material for: Prevalence of central sleep apnea among veterans and response rate to continuous positive airway pressure therapy
Source: Sleep Adv. 2024 Feb 5;5(1):zpae011. doi: 10.1093/sleepadvances/zpae011 (PMC10911693; doi:10.1093/sleepadvances/zpae011)
Supplement: zpae011_suppl_Supplementary_Appendix [file zpae011_suppl_supplementary_appendix.docx]

**Supplement:**

**Appendix 1:**

List of ICD codes used to retrospectively review the Veterans Health Administration (VA) Computerized Patients Record System (CPRS) for codes that include individuals with central sleep apnea and mixed (both obstructive and central) sleep apnea:

G47.30, Sleep apnea, unspecified.

G47.31, Primary central sleep apnea

G47.37, Central sleep apnea in conditions classified elsewhere.

G47.39, Other sleep apnea

**Appendix 2:** Characteristics of the study sample in each subtype of SDB.

| Characteristics | OSA | CSA | COSA |
| --- | --- | --- | --- |
| N  Age (years)  Gender (M/F)  BMI (Kg/m^2^) | 8  67.5 $\pm$11.6  8/0  31.7 $\pm4.0$ | 18  74.0 $\pm$ 9.0  18/0  28.3 $\pm5.7$ | 64  63.4 $\pm$12.9  61/3  31.5 $\pm$ 5.3 |
| Diagnostic AHI  (event/hour)  Diagnostic CAI  (event/hour)  Diagnostic HI  (event/hour)  Diagnostic OAI  (event/hour)  AHI on final PAP level during titration study (PAP0) (event/hour)  CAI on final PAP level during titration study (PAP0) (event/hour) | $61.3 \pm$ 36.8  1.6 $\pm1.6$  35.2 $\pm$27.5  20.8$\pm$ 27.5  9.5 $\pm$ 7.5  1.3 $\pm$ 1.7 | 78.1 $\pm23.2$  59.8 $\pm$ 24.2  9.9 $\pm$8.1  4.2 $\pm$6.6  45.9 $\pm31.1$  11.8 $\pm$ 14.8 | 73.1 $\pm$23.6  16.2 $\pm$ 11.1  39.3 $\pm18$.3  11.2 $\pm$15.0  28.3 $\pm$28.9  $10.8\pm10.8$ |

**Appendix 3:** Characteristics of the study sample based on availability of adherence data to PAP.

| Characteristics | Available PAP adherence data | No PAP adherence data |
| --- | --- | --- |
| N  Age (years)  Gender (M/F)  BMI (Kg/m^2^) | 78  66.7 $\pm$11.6  75/3  31.1 $\pm5.2$ | 12  60.8 $\pm$18.5  12/0  29.2 $\pm$ 6.2 |
| Diagnostic AHI  (event/hour)  Diagnostic CAI  (event/hour)  Diagnostic HI  (event/hour)  Diagnostic OAI  (event/hour)  AHI on final PAP level during titration study (PAP0) (event/hour)  CAI on final PAP level during titration study (PAP0) (event/hour) | $75.3 \pm$ 24.2  22.8 $\pm22.2$  34.0 $\pm$20.8  12.4$\pm$ 16.4  25.8 $\pm$ 26.8*  11.2 $\pm$ 18.5* | 70.1 $\pm$22.0  32.1 $\pm$ 28.9  31.4 $\pm24$.4  3.5 $\pm6$.6  58.3 $\pm33$.4  $37.4\pm32.5$ |

**Definition of abbreviations:** AHI= apnea-hypopnea index, BMI= body mass index, CAI= central apnea index, HI=hypopnea index, OAI=obstructive apnea index, PAP= positive airway pressure, PAP0=PAP titration study. *indicate p<0.05 vs no adherence data using the non-paired Student t-test.
